# Transcranial magnetic stimulation reveals the content of visual short-term memory in the visual cortex

**DOI:** 10.1016/j.neuroimage.2010.01.021

**Published:** 2010-05-01

**Authors:** Juha Silvanto, Zaira Cattaneo

**Affiliations:** a Wellcome Trust Centre for Neuroimaging at UCL, 12 Queen Square, London, UK; b Brain Research Unit, Low Temperature Laboratory, Helsinki University of Technology, Espoo, Finland; c Department of Psychology, University of Milano-Bicocca, Italy

**Keywords:** Imagery, V5/MT+, Transcranial magnetic stimulation, Short-term memory, TMS, Maintenance

## Abstract

Cortical areas involved in sensory analysis are also believed to be involved in short-term storage of that sensory information. Here we investigated whether transcranial magnetic stimulation (TMS) can reveal the content of visual short-term memory (VSTM) by bringing this information to visual awareness. Subjects were presented with two random-dot displays (moving either to the left or to the right) and they were required to maintain one of these in VSTM. In Experiment 1, TMS was applied over the motion-selective area V5/MT+ above phosphene threshold during the maintenance phase. The reported phosphene contained motion features of the memory item, when the phosphene spatially overlapped with memory item. Specifically, phosphene motion was enhanced when the memory item moved in the same direction as the subjects' V5/MT+ baseline phosphene, whereas it was reduced when the motion direction of the memory item was incongruent with that of the baseline V5/MT+ phosphene. There was no effect on phosphene reports when there was no spatial overlap between the phosphene and the memory item. In Experiment 2, VSTM maintenance did not influence the appearance of phosphenes induced from the lateral occipital region. These interactions between VSTM maintenance and phosphene appearance demonstrate that activity in V5/MT+ reflects the motion qualities of items maintained in VSTM. Furthermore, these results also demonstrate that information in VSTM can modulate the pattern of visual activation reaching awareness, providing evidence for the view that overlapping neuronal populations are involved in conscious visual perception and VSTM.

## Introduction

Visual short-term memory (VSTM) is a major process subserved by working memory (see Averbach and Coriell, 1961; Baddeley, 2003; Cornoldi and Vecchi, 2003; Jolicoeur and Dell'Acqua, 1998; Logie, 1995). In VSTM, sensory information is translated into a more durable representation with a duration that outlasts the physical availability of visual input by several seconds (Phillips, 1974). The capacity of VSTM is under debate and it is likely to depend on various factors, such as duration of maintenance, stimulus complexity and the allocation of attentional resources (e.g., Bays and Husain, 2008; Cowan, 2001; Sligte et al., 2008; Stevanovski and Jolicoeur, 2007; Vogel et al., 2001).

The emerging view is that working memory is supported by the coordinated recruitment of brain systems that are involved in processing sensory input and in action-related functions (for a review, see D'Esposito, 2007; Postle, 2006). Specifically, the active maintenance of visual stimuli in working memory is likely to be mediated by the activation of cortical regions that also encode visual input. For example, holding a face in working memory was found to elicit sustained amplitude increases in neurons in face-selective regions of inferotemporal cortex (Chelazzi et al., 1993; Miller and Desimone, 1993. In a similar vein, Serences et al. (2009) demonstrated that activation patterns in V1 during the delay periods of a working memory task reflected the specific attributes that subjects were instructed to maintain: when subjects were remembering the orientation of the sample stimulus, activation patterns in V1 discriminated stimulus orientation, but not stimulus color; the complementary pattern was observed when observers were instructed to remember the color of the stimulus. These results show that early visual areas can retain specific information about visual features held in working memory, over periods of many seconds when no physical stimulus is present (see also Harrison and Tong, 2009). Notably, these activation patterns are specifically tied to the memory delay period, when active rehearsal in working memory is required (Serences et al., 2009; see also Harrison and Tong, 2009). Furthermore, single-cell work has shown that, neurons in the motion-selective area V5/MT+ of the monkey show sustained responses during the delay periods of a memory task requiring to maintain moving stimuli (Bisley et al., 2004). V5/MT+ has also been shown to play a causal role in motion priming, an implicit form of sensory memory (Campana et al., 2002, 2006). Specifically, Campana et al (2006) showed that transcranial magnetic stimulation applied to V5/MT+ abolishes priming for motion direction (see also Cattaneo et al., 2009b).

Here we made use of TMS state-dependency (cf Silvanto and Muggleton, 2008a,b) to explore the information content of specific visual cortical regions during VSTM maintenance. Specifically, we investigated whether activity in a specific cortical area, the motion-selective region V5/MT+, reflects the motion attributes of the memory item, and whether TMS can bring these attributes to visual awareness. The latter would be direct causal evidence for the view that the same neuronal representations are involved in both VSTM maintenance and conscious perception of a given perceptual attribute. TMS-induced phosphenes were used to assess visual cortical information content. An advantage of phosphenes is that they provide a qualitative, perceptual measure of TMS-induced activation and thus complement psychophysical measures in which the nature of TMS effects are inferred from effects on objective measures such as reaction times and detection accuracies (Marg and Rudiak, 1994). Phosphenes have been used to investigate the neural impact of a wide-range of processes such as attention (Bestmann et al., 2007; Cattaneo et al., 2009a), auditory–visual interactions (Romei et al., 2009; Romei et al., 2007), visual imagery (Sparing et al., 2002) and cortico-cortical interactions within the visual system (e.g. Pascual-Leone and Walsh, 2001; Silvanto et al., 2006, 2009). We made use of the earlier finding that TMS applied during VSTM maintenance strengthens the representation of the memory item, indicating that the excitability of neurons engaged in maintenance is increased (Cattaneo et al., 2009b). The implication of this TMS state-dependency (Cf. Silvanto & Muggleton, 2008a,b) is that if neurons in a given visual area are engaged in VSTM, then phosphenes induced from this region during VSTM maintenance should reflect the properties of the memory item because neurons engaged in maintenance are more readily activated.

We thus investigated whether phosphenes induced from V5/MT+ reflect the motion qualities of a memory item containing visual motion. Furthermore, we investigated the possibility that any such effect occurs only when the induced phosphene spatially overlaps with the memory item. On each trial, subjects were asked to maintain a motion stimulus in VSTM and a phosphene was induced during the maintenance period. Phosphene appearance was assessed using a subjective rating scale (for a similar procedure, see Pascual-Leone and Walsh, 2001; Silvanto et al., 2005). In order to ensure that subjects were actively engaged in maintenance, they were asked to judge, at the end of each trial, whether a test stimulus moved slower or faster than the memory item. In Experiment 1, phosphenes were induced from left and right V5/MT+; in Experiment 2, we investigated the generality of any effect found in Experiment 1 by investigating whether such effects are found also in phosphenes induced from regions not implicated in the maintenance of simple motion information. In this experiment, TMS was applied over the left and right lateral occipital region.

## Methods

### Experiment 1

#### Subjects

Twelve subjects (8 males and 4 females, aged 18 to 35 years) were recruited for the experiment. An inclusion criterion was the ability to perceive moving phosphenes and three subjects were excluded for this reason. All subjects gave informed consent before participating in the study which had been approved by the local ethics committee and were treated in accordance with the declaration of Helsinki.

#### TMS stimulation and site localisation

TMS was delivered by means of two Magstim Super Rapid machines (Magstim, UK) via 70 mm figure-of-eight-shaped coils. The sites for stimulation were located using a functional method typically used in studies investigating phosphenes (Stewart et al., 1999, see Walsh and Pascual-Leone, 2003, for a detailed discussion). The left and right V5/MT+ were localised by the production of moving phosphenes; a technique that has been used in a number of studies on phosphenes and V5/MT+ function (e.g. Campana et al., 2002; Silvanto et al., 2005) and which provides a localisation consistent with fMRI localisers (Thompson et al., 2009). In this functional localisation technique, phosphenes are induced from each point in a 3 × 3-cm grid around a central point of 3 cm dorsal and 5 cm lateral from the inion. The location from which the most vivid moving phosphenes were induced was used in the main experiment. In all subjects, phosphene motion was away from the midline (either moving horizontally towards the periphery or at an angle towards the lower visual field). Drawings of phosphenes were obtained from each subject to confirm overlap with the visual stimulus location in the VSTM task (see below for description of the task). The average coil positions for left V5/MT+ was 3.2 cm dorsal and 5.3 cm lateral from the inion and for the right V5/MT+ 3.1 cm dorsal and 5.4 cm lateral from the inion. Coil orientation was such that the handle was pointing medial to lateral away from the midline. Stimulation site localisation as well as the main experiment took place in a darkened room. For each subject, the phosphene threshold was identified using a modified binary search algorithm (Tyrrell and Owens, 1988). The mean phosphene thresholds were 68% and 70% (of maximum stimulator output) for left and right V5/MT+, respectively. To ensure that subjects would perceive phosphenes on most of the trials, TMS was applied at an intensity of 20% above phosphene threshold in the main experiment. Throughout the main experiment, two coils were placed on the subject's head, one over the left V5/MT+ and the other over the right V5/MT+. Subjects were asked to provide ratings of phosphene appearance using the following scale: 0 = phosphene was absent; 1 = phosphene was stationary; 2 = subject was uncertain whether phosphene was moving or stationary; 3 = the phosphene was moving in a specific direction (and if so, which direction) (for a similar procedure, see Pascual-Leone and Walsh, 2001).

#### Visual stimuli

The motion stimuli (presented with a 17-inch (800 × 600) CRT monitor) appeared within an imaginary square that subtended 10° by 10° of visual angle at a viewing distance of approximately 57 cm. The stimuli consisted of 200 white dots (with a size of 1 pixel each), placed at random positions on a black background. The dots moved at a speed of either 2 or 3 pixels per frame (frame duration: 16 ms). The motion direction was either to the left or right. The stimuli were presented in the lower quadrant of either left or right hemifield, with center of the stimulus at an eccentricity of 10° of visual angle from both the vertical and horizontal meridian (as this was the location in the visual field where the phosphenes most frequently appeared).

#### Visual short-term memory (VSTM) task

**Fig. 1:**
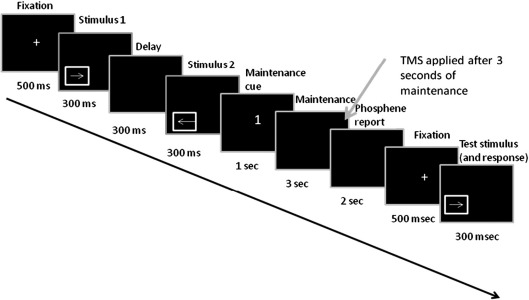
Timeline of an experimental trial. On each trial, subjects were presented with two motion stimuli; on “maintenance” trials, they were asked to hold either the first or second stimulus in short-term memory. During the maintenance period, a phosphene was induced from either the left or right V5/MT+ (Experiment 1) or from left or right lateral occipital region (LO). Subjects were asked to verbally provide a rating of their phosphene. They were then presented with a test stimulus and asked to judge whether it moved at a slower or faster speed than the memory item.

Fig. 1 shows the timeline of each experimental trial. Each trial began with a fixation cross appearing for 500 ms. Subjects were then presented with two motion stimuli (with a duration of 300 ms each) in rapid succession, with an inter-stimulus interval of 300 ms. The two motion stimuli differed in their motion direction, with all the dots in the motion stimuli moving either to the left or to the right, but the speed was constant. The two stimuli were presented in the same quadrant. On half of the trials, both stimuli appeared within the lower left quadrant and on the other half they both appeared within the lower right quadrant.

The motion stimuli were followed by a display (1000 ms) indicating whether the subject should hold in their memory the speed and direction of the first stimulus (indicated by the number “1”), the second stimulus (“2”) or no stimulus (“0”). After a 3-s delay (during which subjects were asked to maintain a mental image of the memory item), a TMS pulse was applied either over the left or right V5/MT+ (at the start of the experiment, subjects were informed that on each trial, either hemisphere could be stimulated). After the TMS pulse, subjects were asked to verbally provide (within a 2-s response period), a rating of phosphene appearance using the phosphene scale of Pascual-Leone and Walsh (2001). After the response period, a fixation cross (500 ms) appeared in the center of the screen, followed by a motion stimulus (300 ms) moving in the same direction but either at a slower or faster speed than the memory item (speed difference was 1 pixel per frame) and presented in the same quadrant as the memory item. Subjects were asked to report whether this stimulus was moving at a faster or slower speed than the memory item. This speed discrimination task was used in order to prevent the use of verbal cues (such as “left” or “right”) that could have been used in case of a direction discrimination task during the maintenance phase. Moreover, as there was an equal probability of the test stimulus moving at a slower or faster speed than the memory item, this task could not be performed by assigning a verbal tag such “slow” or “fast” to the memory item. Rather, accurate performance required an active maintenance of motion speed.

#### Trial types

There were a total of 128 “Maintenance and TMS” trials, i.e. trials on which subjects were asked to maintain a motion stimulus (and perform the VSTM task at the end of the trial), and on which TMS was applied over either the left or right V5/MT+ (64 trials for both the left and right V5/MT+). Whether the motion stimuli at the start of the trial appeared in the left or right lower quadrant, and whether subjects were asked to maintain the first or second motion stimulus in VSTM, was counterbalanced. There were a total of 64 “spatially congruent” (i.e. when the phosphene spatially overlapped with the location of the memory item) and 64 “spatially incongruent” (i.e. when the phosphene appeared contralateral to the memory item) TMS trials.

There were a total of 64 “No maintenance and TMS” trials. On these trials, subjects were informed by a cue *after* the presentation of the two motion stimuli that no maintenance was required (and that the VSTM task would not be performed). The maintenance cue was presented *after* stimulus presentation to ensure that there would be no fundamental differences in the encoding of the target stimulus in the “Maintenance” and “No maintenance” trials.

There were a total of 64 “Baseline phosphene” trials on which no visual motion stimuli were presented before phosphene induction. These trials were used as the baseline measure for subjects' phosphene.

There were a total of 32 “Maintenance and No TMS” trials on which subjects performed the VSTM task but no TMS was applied. This condition was included in order to be able to determine the possible impact of TMS on the VSTM task.

#### Procedure

At the start of the session, subjects performed a block of 32 trials on the VSTM task without any TMS to obtain a measure of their performance without TMS (this block was preceded by a practice block of 12 trials). The left and right V5/MT+ were then localised. At this stage, three subjects who could not perceive moving phosphenes were excluded from the study. The 64 “baseline phosphene” trials were carried out in two blocks of 32 trials (one block was run immediately after V5/MT+ localisation and the other at the end of the experiment). The rest of the conditions were run in eight blocks of 24 trials. All the blocks contained 16 “Maintenance and TMS” trials and 8 “No maintenance and TMS” trials. The trials were counterbalanced in such a way that in each block, on half of the trials the left V5/MT+ was stimulated and on the other half the right V5/MT+ was stimulated. The location of the motion stimulus (i.e. whether it appeared on the left or right lower quadrant) was also counterbalanced.

### Experiment 2

A further experiment was performed to investigate whether any impact of VSTM maintenance on phosphene perception is restricted to phosphenes induced from the motion-selective V5/MT+ region, or whether phosphenes induced from regions which are not motion- selective are also affected, possibly reflecting a general effect of VSTM maintenance on visual perception. This possibility was tested by examining the impact of VSTM maintenance on phosphenes induced from the left and right lateral occipital cortex, a region associated with object processing ( e.g. Grill-Spector, 2003; Ishai et al., 1999).

As previously done by Ellison and Cowey (2006), the lateral occipital (LO) region was located in relation to V5/MT+. Specifically, LO was calculated to be 1.5 cm caudal on the skull in a direct line towards the inion in accordance with various anatomical and functional maps (Van Essen et al., 2001). As with V5/MT+ TMS, coil orientation was such that the handle was pointing medial to lateral away from the midline. All subjects perceived phosphenes from both the left and right LO region. These phosphenes were large (similar to size to V5/MT+ phosphenes) and appeared in the contralateral lower hemifield (thus overlapping with the stimulus location to similar extent as V5/MT+ phosphenes). In all subjects, these phosphenes were stationary. Drawings of phosphenes were obtained from each subject to confirm overlap with the visual stimulus location in the VSTM task. For each subject, the phosphene threshold was identified using a modified binary search algorithm (Tyrrell and Owens, 1988). The mean phosphene thresholds were 71% and 75% of maximum stimulator output for left and right LO, respectively. The experimental task and procedure were identical to that of Experiment 1.

## Results

### Experiment 1

At first we examined whether the mere presentation of motion stimuli (without any maintenance) affected subjects' phosphene judgments. This was accomplished by comparing phosphene ratings in the “No maintenance and TMS” condition (i.e. trials on which subjects were presented with the motion stimuli but were not asked to perform the maintenance task) to phosphene ratings in the “baseline phosphene” condition (i.e. trials on which no visual motion stimuli were presented before phosphene induction).

This analysis revealed no significant differences, regardless of whether the phosphene appeared in the same spatial location as the memory item (*t*(8) = 0.43; *p* = 0.68) or whether the phosphene appeared in the other hemifield (*t*(8) = 0.41; *p* = 0.69). The mean phosphene ratings were as follows: Baseline phosphene, 2.09; Phosphene location spatially overlapping with the memory item, 2.14; and Phosphene location not spatially overlapping with the memory item, 2.12.

#### The impact of VSTM maintenance on phosphene ratings

For trials involving VSTM maintenance, only trials on which subjects performed correctly in the speed discrimination task were included in the analysis. The maintenance conditions were divided into the following categories as a function of (a) whether the motion direction of the memory item was congruent with the direction of phosphene motion (note that phosphene motion in all subjects was away from the fovea; therefore, the *congruent direction* was always away from the fovea and the *incongruent direction* was always towards the fovea); and b) whether the memory item spatially overlapped with the phosphene location. The “maintenance and TMS” trials were thus divided into four types:

1) Memory item motion direction congruent with baseline phosphene; spatial overlap.
2) Memory item motion direction congruent with baseline phosphene; no spatial overlap.
3) Memory item motion direction incongruent with baseline phosphene; spatial overlap.
4) Memory item motion direction incongruent with baseline phosphene; no spatial overlap.

**Fig. 2:**
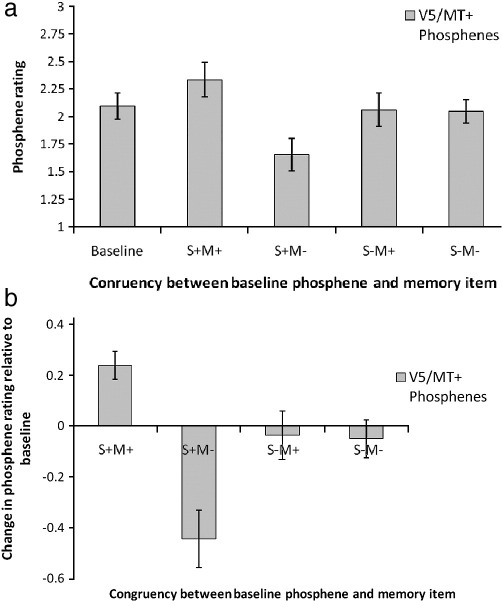
Mean (*n* = 9) of subjects' phosphene ratings in Experiment 1. Subjects were asked to provide ratings of phosphene appearance using the following scale: 0 = phosphene was absent; 1 = phosphene was stationary; 2 = subject was uncertain whether phosphene was moving or stationary; 3 = the phosphene was moving in a specific direction (and if so, which direction). This phosphene scale is adapted from Pascual-Leone and Walsh (2001). (a) The “maintenance and TMS” trials were divided into four categories as a function of whether the memory item (a) spatially overlapped with the V5/MT+ phosphene location and (b)whether the motion direction of the memory item was the same as in the V5/MT+ baseline phosphene (i.e. away from the center). The conditions are abbreviated in the following way: S+M+: spatially overlapping, motion direction congruent; S+M−: spatially overlapping, motion direction incongruent; S−M+: spatially non-overlapping, motion direction congruent; S−P−: spatially non-overlapping, motion direction incongruent. Relative to the baseline condition, phosphene appearance was only affected when the phosphene spatially overlapped with the memory item (conditions S+M+ and S+M−). The appearance of phosphene motion was enhanced when the memory item moved in the same direction as the baseline phosphene; phosphene motion was reduced when the memory item moved in the opposite direction than the baseline phosphene. The error bars indicate ± 1 SEM. (b) The relative impact of VSTM maintenance on phosphene ratings. This figure shows the mean (*n* = 9) phosphene rating on each type of “maintenance and TMS” trial *subtracted* from the baseline phosphene rating.

Fig. 2a shows the mean of subjects' phosphene ratings for each of these categories. A repeated-measures ANOVA with trial type as the main factor (with five levels: the four trial types and “baseline”) indicated a significant main effect (*F*(4) = 18.93 *p* = 0.0001). Pairwise comparisons revealed that, relative to the baseline condition, phosphene ratings were affected when the memory item and the phosphene spatially overlapped. Specifically, in the memory item/phosphene spatial overlapping condition, there was a decrease in phosphene rating relative to the baseline measure (i.e. phosphene ratings in the control runs) when the motion direction of the memory item was *incongruent* with the direction of phosphene motion (*t*(8) = 4.39; *p* = 0.002). This indicates that subjects reported their phosphene to contain motion less frequently than was the case in the baseline condition. In contrast, there was an *increase* in the phosphene rating when the direction was *congruent* with the direction of phosphene motion (*t*(8) = 3.95; *p* = 0.004). This in turn indicates that the subjects reported their phosphene to contain motion more frequently than in the baseline condition.

When there was no spatial overlap between the memory item and the phosphene, subjective ratings were not significantly different from the baseline condition (*Direction congruent; no spatial overlap*: *t*(8) = 0.36; *p* = 0.72; *Direction incongruent; no spatial overlap*: *t*(8) = 0.66; *p* = 0.53). Fig. 2b shows the relative impact of VSTM maintenance on phosphene ratings by showing the phosphene rating on each type of maintenance trial subtracted from the baseline phosphene rating.

The number of trials on which subjects did not perceive a phosphene (i.e. the phosphene rating was 0) in each of the four trial types (described on page 9) were Trial type 1: 2; Trial type 2: 3.3; Trial type 3: 2.9; and Trial type 4: 3.6. In the control condition, in which subjects did not engage in maintenance, there were on average 2.9 of such trials.

Fig. 2b suggests that the decrease in the phosphene rating relative to baseline for the spatially overlapping incongruent condition is a much larger effect than the increase in rating for the equivalent congruent condition. However, this difference is not statistically significant (*t*(8) = 1.45; *p* = 0.18).

The mean accuracies in the VSTM task as a function of trial type were *Direction congruent, no spatial overlap*: 84%; *Direction incongruent, no spatial overlap*: 87%; *Direction congruent, spatial overlap*: 85%; and *Direction incongruent, spatial overlap*: 79%. Subjects' mean accuracy in the VSTM task in the absence of TMS (*No TMS condition*) was 89%. A repeated-measures ANOVA with trial type as the main factor revealed a significant main effect (*F*(4) = 3.01; *p* = 0.03). Pairwise comparisons indicated that, relative to the No TMS condition, VSTM performance was impaired in the “Direction incongruent; spatial overlap” condition (*t*(8) = 2.75; *p* = 0.025). Furthermore, performance in the “Direction congruent; spatial overlap” condition was significantly higher than in the “Direction incongruent; spatial overlap” condition (*t*(8) = 3.25; *p* = 0.011). Other comparisons were not statistically significant.

### Experiment 2

As in the analysis of Experiment 1, at first we examined whether the mere presentation of motion stimuli (without any maintenance) affected subjects' phosphene judgments. This was accomplished by comparing phosphene ratings in the “No maintenance and TMS” condition (i.e. trials on which subjects were presented with the motion stimuli but were not asked to perform the maintenance task) to phosphene ratings in the “baseline phosphene” condition (i.e. trials on which no visual motion stimuli were presented before phosphene induction).

For LO phosphenes, this analysis revealed no significant differences between the conditions, regardless of whether the phosphene appeared in the same spatial location as the memory item (*t*(8) = 0.19; *p* = 0.85) or whether the phosphene appeared in the other hemifield (*t*(8) = 0.46; *p* = 0.66). The mean phosphene ratings were as follows: Baseline phosphene, 0.97; Phosphene location spatially overlapping with the memory item, 0.98; and Phosphene location not spatially overlapping with the memory item, 0.99.

#### The impact of VSTM maintenance on phosphene ratings

For trials involving VSTM maintenance, only trials on which subjects performed correctly in the speed discrimination task were included in the analysis. The maintenance conditions were divided into two categories as a function of whether or not the phosphene spatially overlapped with the memory item. Because LO baseline phosphenes do not contain motion, the trials could not be divided as a function of whether the motion component of the baseline phosphene was congruent or incongruent with the motion direction of the memory item. There were thus two trial types:

1) Spatial overlap between the phosphene and the memory item.
2) No Spatial overlap between the phosphene and the memory item.

**Fig. 3:**
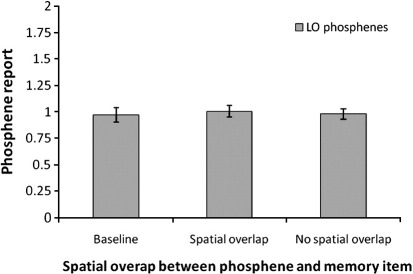
Mean (*n* = 9) phosphene ratings in Experiment 2. The “maintenance” trials were divided into two categories as a function of whether or not the memory item spatially overlapped with the location of the phosphene induced from the lateral occipital region. Phosphene appearance was not affected by VSTM maintenance. The error bars indicate ± 1 SEM.

Fig. 3 shows the mean of subjects' phosphene ratings for phosphenes induced from LO. A repeated-measures ANOVA with trial type as the main factor (with three levels: the two trial types and “baseline”) indicated no significant main effect (*F*(2) = 0.16; *p* = 0.85), indicating that LO phosphene were not influenced by the motion qualities of the memory item.

The mean accuracies in the VSTM task as a function of trial type were as follows: spatial overlap, 88%; no spatial overlap, 86%. Subjects' performance in the VSTM task in the absence of TMS was 91%. A repeated-measures ANOVA found no significant differences between the conditions (*F*(2) = 1.21; *p* = 0.32).

## Discussion

Our results show VSTM maintenance of motion information influences the activation reaching awareness from the motion-selective extrastriate area V5/MT+, demonstrating that activity in V5/MT+ reflects the motion qualities of items maintained in VSTM. Furthermore, that TMS could transfer information from VSTM to conscious perception suggests that these two processes engage the same neuronal representations. In Experiment 1, where phosphenes were induced from V5/MT+ during the maintenance period, the phosphene motion was influenced by the motion component of the memory item. Specifically, phosphene motion was enhanced when motion direction in the memory item was the same as in the baseline V5/MT+ phosphene (i.e. motion away from the fovea); in contrast, phosphene motion was weakened when the motion in the memory item was to the opposite direction as what was contained in the baseline V5/MT+ phosphene (i.e. motion towards the fovea). The nature of this maintenance/phosphene appearance interaction demonstrates that V5/MT+ is involved in the maintenance of motion information. Importantly, these interactions only occurred when the phosphene spatially overlapped with the memory item, demonstrating that the VSTM holds information in a retinotopic fashion.

In Experiment 2, phosphenes induced from the lateral occipital region were not affected by phosphene motion even when there was a spatial overlap between the phosphene and the memory item. This is an expected finding, given the neuroimaging evidence that motion processing does not specifically engage the LO region unless the motion represents a cue for object recognition (Ferber et al., 2003; Grill-Spector et al., 1998; Kourtzi and Kanwisher, 2001). The lack of an effect in Experiment 2 demonstrates that VSTM maintenance of motion information does not affect all visual cortical activation reaching awareness; rather, the effect seems to be specific to regions which are engaged in VSTM maintenance.

Our findings are consistent with the sensory recruitment hypothesis, according to which the neural mechanisms that are involved in the short-term storage of visual information are also closely associated with those areas that are involved in its sensory analysis (Bisley and Pasternak, 2000; Fuster, 1997; Gibson and Maunsell, 1997; Pasternak and Greenlee, 2005). Recent fMRI studies (Harrison and Tong, 2009; Serences et al., 2009; Pearson et al., 2008) have provided strong evidence in support of this view by demonstrating that the content of VSTM can be decoded from the activity of a “low-level” sensory area such as V1. Our study contributes to this literature by providing evidence that V5/MT+ activation reflects the content of information maintained in VSTM. Importantly, our results also show that TMS can bring features of an internal mental image to visual awareness.

That TMS seems to preferentially activate neurons engaged in VSTM maintenance (and facilitate VSTM performance, cf. Cattaneo et al., 2009b) might appear surprising given that TMS has the opposite, disruptive effect when applied in visual motion priming paradigms (Campana et al., 2002, 2006). Both visual priming and VSTM are based on visual cortical activation outlasting the presentation of a visual stimulus and one might therefore expect them to rely on similar neural processes. There is an important difference however: VSTM tasks require *active maintenance* of visual information whereas this is not the case in the visual priming paradigms previously used in conjunction with TMS. Recent fMRI evidence suggests that this difference is important: in an fMRI study by Soto et al (2007), the reappearance of a stimulus held in working memory enhanced activity in occipital areas that are known to encode the prior occurrence of stimuli. In contrast, mere stimulus repetition elicited a suppressive response in the same regions. That TMS induces opposite effects in “active” VSTM maintenance and “passive” priming paradigms is consistent with the evidence of Soto et al (2007) that different neural states associated with stimuli held in working memory and passively observed targets.

The shape, motion and intensity of TMS-induced phosphenes have been used to investigate questions such as the role of feedback connections in visual awareness (Pascual-Leone and Walsh, 2001; Silvanto et al., 2005), neural basis of visual imagery and attention (Bestmann et al., 2007; Sparing et al., 2002) and auditory–visual cortical interactions (Romei et al., 2009; Romei et al., 2007). As with any subjective report, it is important to rule out the possibility that these effects do not reflect demand characteristics or simple response heuristics which subjects may adopt. There are number of reasons why it is unlikely that in the present study subjects were simply using the motion direction of the memory item as a heuristic in their phosphene report. Firstly, phosphene appearance was only affected when the phosphene spatially overlapped with the memory item; such a pattern of results would not have been observed if these results merely reflected a non-perceptual bias to report features of the memory item. Secondly, ratings of LO phosphenes were unaffected by the memory item, even when the LO phosphene spatially overlapped with the memory item. Finally, the present study used an established phosphene report procedure (cf. Pascual-Leone and Walsh, 2001). It is also important to note that V5/MT+ phosphenes were unaffected by the motion stimuli when subjects were not asked to engage in VSTM maintenance.

In summary, our results demonstrate that V5/MT+ activity reflects the motion attributes of stimuli held in VSTM. Furthermore, that TMS could bring these motion attributes to conscious perception indicates that VSTM and visual awareness rely on the same neuronal representations.
